# Basal Forebrain Atrophy Is Associated With Allocentric Navigation Deficits in Subjective Cognitive Decline

**DOI:** 10.3389/fnagi.2021.596025

**Published:** 2021-02-15

**Authors:** Qian Chen, Sichu Wu, Xin Li, Yi Sun, Wenqian Chen, Jiaming Lu, Wen Zhang, Jiani Liu, Zhao Qing, Zuzana Nedelska, Jakub Hort, Xin Zhang, Bing Zhang

**Affiliations:** ^1^Department of Radiology, Drum Tower Hospital, Clinical College of Nanjing Medical University, Nanjing, China; ^2^Department of Radiology, Drum Tower Hospital, Medical School of Nanjing University, Nanjing, China; ^3^Institute of Brain Science, Nanjing University, Nanjing, China; ^4^Memory Clinic, Department of Neurology, 2nd Faculty of Medicine, Charles University, University Hospital Motol, Prague, Czechia; ^5^International Clinical Research Center, St. Anne's University Hospital Brno, Brno, Czechia

**Keywords:** subjective cognitive decline, basal forebrain, entorhinal cortex, spatial navigation, allocentric

## Abstract

Individuals with subjective cognitive decline (SCD) are at higher risk of incipient Alzheimer's disease (AD). Spatial navigation (SN) impairments in AD dementia and mild cognitive impairment patients have been well-documented; however, studies investigating SN deficits in SCD subjects are still lacking. This study aimed to explore whether basal forebrain (BF) and entorhinal cortex (EC) atrophy contribute to spatial disorientation in the SCD stage. In total, 31 SCD subjects and 24 normal controls were enrolled and administered cognitive scales, a 2-dimensional computerized SN test, and structural magnetic resonance imaging (MRI) scanning. We computed the differences in navigation distance errors and volumes of BF subfields, EC, and hippocampus between the SCD and control groups. The correlations between MRI volumetry and navigation distance errors were also calculated. Compared with the controls, the SCD subjects performed worse in both egocentric and allocentric navigation. The SCD group showed volume reductions in the whole BF (*p* < 0.05, uncorrected) and the Ch4p subfield (*p* < 0.05, Bonferroni corrected), but comparable EC and hippocampal volumes with the controls. In the SCD cohort, the allocentric errors were negatively correlated with total BF (*r* = −0.625, *p* < 0.001), Ch4p (*r* = −0.625, *p* < 0.001), total EC (*r* = −0.423, *p* = 0.031), and left EC volumes (*r* = −0.442, *p* = 0.024), adjusting for age, gender, years of education, total intracranial volume, and hippocampal volume. This study demonstrates that SN deficits and BF atrophy may be promising indicators for the early detection of incipient AD patients. The reduced BF volume, especially in the Ch4p subfield, may serve as a structural basis for allocentric disorientation in SCD subjects independent of hippocampal atrophy. Our findings may have further implications for the preclinical diagnosis and intervention for potential AD patients.

## Introduction

Alzheimer's disease (AD), a global concern, is a progressive neurodegenerative disorder that contains three stages: the preclinical stage, mild cognitive impairment (MCI), and dementia (Sperling et al., 2011). Subjective cognitive decline (SCD), a self-perceived worsening of cognitive function without objective deficits in neuropsychological evaluations, is considered to be a clinically-based approach for the detection of subjects at a potentially higher risk of developing AD (Jessen et al., 2014, 2020). SCD corresponds to the preclinical stage of the AD spectrum; thus, it is of critical importance to fully investigate features and biomarkers of this stage to pave the way for early diagnosis and intervention in AD (Howard, 2020; Jessen et al., 2020).

It has been well-established by histopathological studies that AD is associated with the loss of cholinergic neurons (Davies and Maloney, 1976; Mcgeer et al., 1984). Treatment with cholinesterase inhibitors has proven effective in improving global cognitive function, the activities of daily living, and behavioral symptoms in patients with mild to moderate AD (Raskind et al., 2000; Tariot et al., 2000; Rockwood et al., 2006). The basal forebrain (BF), consisting of different subfields such as Ch1-4, is a key structure for cholinergic input to the hippocampus, amygdala, and cerebral cortex (Mesulam et al., 1983). Studies based on magnetic resonance imaging (MRI) volumetry have shown significant volume reductions of the BF in MCI and AD dementia patients (Teipel et al., 2011; Grothe et al., 2012, 2013). The reduced volumes in specific subfields correlated with impairments in different cognitive domains (Grothe et al., 2010). However, to our knowledge, only one recent study has reported Ch4p volume reductions in the BF in a cohort of 24 SCD subjects (Scheef et al., 2019).

The entorhinal cortex (EC) is recognized as one of the earliest affected regions by AD pathology, and previous studies have shown cortical thinning and volume reductions in the EC in SCD subjects (Jessen et al., 2006; Meiberth et al., 2015; Ryu et al., 2017). Furthermore, a longitudinal study using the Alzheimer's Disease Neuroimaging Initiative (ADNI) cohort revealed that BF atrophy preceded entorhinal volume reduction and could predict the cortical spread of AD pathology and memory impairments in MCI patients (Schmitz et al., 2016).

Patients with MCI and AD dementia experience difficulties with spatial navigation (SN), which is the ability to determine and maintain a route from one place to another (Hort et al., 2007; Nedelska et al., 2012; Lithfous et al., 2013). Two SN strategies have been well-established: egocentric navigation and allocentric navigation (O'Keefe and Nadel, 1978). Egocentric navigation relies on subject-to-object relations and leads to the constitution of self-centered representations, while allocentric navigation depends on object-to-object relations and contributes to the construction of world-centered representations (Colombo et al., 2017). Lesion studies in mice have provided direct evidence that BF lesions result in both egocentric and allocentric disorientation (Berger-Sweeney et al., 2001; Hamlin et al., 2013). Previous studies have shown that BF atrophy was associated with allocentric impairments in AD patients (Kerbler et al., 2015b). Furthermore, treatment with donepezil, a cholinesterase inhibitor, has suggested improved performance in allocentric but not egocentric navigation in AD patients (Hort et al., 2014). The EC contains grid cells, which show a six-fold modulated firing pattern and play a critical role in allocentric representations (Hafting et al., 2005; Doeller et al., 2010). However, whether BF and EC atrophy contribute to SN deficits in SCD subjects remains unresolved.

In the present study, we aimed to determine the alterations in volumes of BF subfields and the bilateral EC in SCD subjects and to further elucidate the associations between MRI volumetry and navigation performance assessed by a 2-dimensional computerized SN test. We hypothesized that SCD individuals would show reduced volumes in the BF, most pronounced in the Ch4p subregion, and reduced volumes in the EC compared to the control subjects. Consistent with previous studies, we also expected significant associations between structural measures and allocentric navigation performance, which may indicate the structural neural basis of allocentric navigation deficits in SCD subjects.

## Materials and Methods

### Subjects

Fifty-six individuals with Chinese Han nationality were recruited from the Drum Tower District of Nanjing by advertisement, and one subject showing bad homogeneity of imaging data was excluded. In total, 55 subjects were enrolled in the present study. The inclusion criteria were 55–75 years old, right-handedness, and equal to or more than 9 years of education experience. Participants with a history of stroke, other neurological disorders that could lead to cognitive decline (Parkinson's disease, encephalitis, epilepsy, brain tumors, etc.), severe anxiety or depression, and contraindications for MRI scanning were excluded from the study. Subjects who met the diagnostic for MCI in the standardized neuropsychological evaluation were also excluded from the current study. Specifically, three cognitive domains each containing two subtests were assessed: Auditory Verbal Learning Test (AVLT) long-delayed memory and AVLT recognition (Zhao et al., 2012) for episodic memory; Trail Making Test Part A (TMT-A) and Part B (TMT-B) (Zhao et al., 2013) for executive function; and Boston Naming Test (BNT) (Mack et al., 1992) and Animal Fluency Test (AFT) (Henry et al., 2004) for language ability. Participants were considered MCI patients with scores >1 standard deviation (SD) below the normative means in both subtests within one cognitive domain or >1 SD below the normative means in three single tests in three different domains (Jak et al., 2009; Li et al., 2019). The participants were assigned to the SCD group if they complained of memory decline within the last 5 years and expressed worries associated with memory decline. In total, 31 subjects were assigned to the SCD group. Twenty-four age-, sex-, and education-matched old people without memory complaints and cognitive impairments were recruited as normal controls (NCs). All participants signed an informed consent statement after gaining a sufficient understanding of the study procedures. The experiment was approved by the Medical Research Ethics Committee of Nanjing Drum Tower Hospital.

### Neuropsychological Evaluation

Each participant completed a set of standardized neuropsychological tests. The cognitive evaluation was performed by a psychologist with 10 years of working experience. The Mini-Mental State Examination (MMSE) (Tombaugh and Mcintyre, 1992) was implemented to measure global cognition, and the SCD questionnaire (SCD-Q) was employed for a quantitative assessment of the severity of SCD (Supplementary Box 1) and was not the inclusion criteria for SCD (Gifford et al., 2015; Li et al., 2019). Except for AVLT, TMT-A, TMT-B, BNT, and AFT mentioned above, we also used the Rey-Osterrieth Complex Figure (ROCF) (Shin et al., 2006) recall test to measure visuospatial memory, ROCF copy test and the Clock Drawing Test (CDT) (Shulman, 2000) to assess visuospatial abilities, and the Symbol Digit Modalities Test (SDMT) (Sheridan et al., 2006) to evaluate processing speed. The measures from the TMT-A and TMT-B tests are reported as the time (in seconds) spent on the test, with longer times representing worse executive function. Higher scores in the SCD-Q suggest worse self-assessment of cognition. For the other cognitive tests, measures are reported as the numbers of correct responses, with higher scores reflecting better function in the corresponding cognitive domains.

### Spatial Navigation Assessment

**Figure 1:**
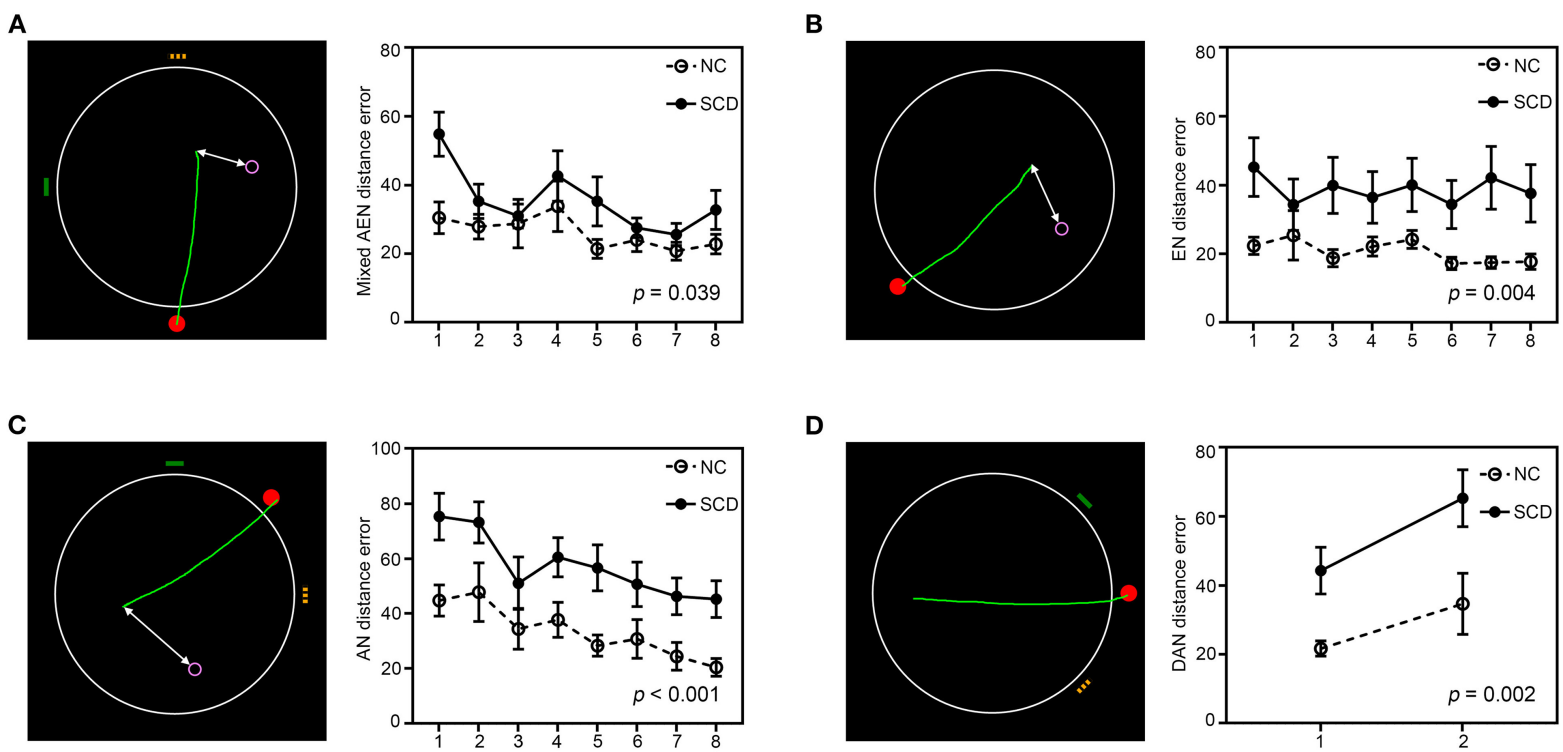
The 2-dimensional computerized hidden goal task and corresponding navigation distance errors in each subtest. The images show an aerial view of the arena (large white circle), the starting point (red filled circle), orientation cues (yellow and green lines), and the goal (purple hollow circle). The green lines represent tracking by a subject from the start point to the supposed goal position, and the white lines represent the distance errors. Navigation distance errors in the normal control (NC) and subjective cognitive decline (SCD) groups in each trial of the **(A)** mixed alloegocentric navigation subtest (AEN), **(B)** egocentric navigation subtest (EN), **(C)** allocentric navigation subtest (AN), and **(D)** delayed allocentric navigation subtest (DAN) are shown. Values are the mean ± SEM (For interpretation of the references to colors in this figure, the reader is referred to the web version of this article).

The navigation behavior was measured by the Amunet test battery (NeuroScios, Austria, Gmbh), a computer-based version of the Morris water maze (hMWM), which used a similar paradigm as the hidden goal task (Kalová et al., 2005; Hort et al., 2007; Nedelska et al., 2012). Participants were presented with a computer screen (640 ×480 pixels) that showed a large white circle with 280 pixels in diameter representing the overhead view of the arena. Briefly, a red dot was the starting point, and yellow and green lines on the edge of a large white circle were the orienting cues. A purple hollow circle with 16 pixels in diameter was the goal, which was shown at the beginning and then disappeared in each trial. The examinee was asked to draw a path from the start to the goal as accurately as possible using a mouse. After the subject indicated the supposed goal position, the correct position was shown and the subject again was encouraged to notice its relative position to the starting point or cues. The task contained four phases from simple to complex: (a) Mixed alloegocentric navigation (Figure 1A): The least demanding subtask, which was considered a training task designed to get familiarized with the SN test. The examinee could find the goal by its spatial relationship with both the starting point and the orienting cues. (b) Egocentric navigation (Figure 1B): The examinee could locate the hidden goal only by its mutual relationship with the starting point, as the orienting cues were not displayed on the screen. (c) Allocentric navigation (Figure 1C): The examinee could locate the hidden goal using only its relationship with the orienting cues, as the position of the starting point was unrelated to the goal. (d) Delayed allocentric navigation (Figure 1D): This subtest was performed 30 min later using the same strategy as allocentric navigation to measure the delayed recall ability, during which the correct goal position was not shown so as to prevent the subjects from learning (Laczó et al., 2011). It is analogous to the probe trial in the original MWM task, where the hidden platform is removed and only distal orientation cues are used for navigation (Laczó et al., 2015). There were eight trials each of the mixed alloegocentric, egocentric, and allocentric subtests, while there were two trials of the delayed allocentric subtest. SN performance was recorded automatically as the average distance errors (from the position drawn by the examinee to the correct position of the goal on the computer screen in pixels) across all trials of each subtest. The SN task was not time-restricted to reduce bias by differences in cognitive, sensory, and physical functioning (Laczo et al., 2014). The examiner was blind to the diagnosis. Two SCD participants did not complete the delayed subtest; thus, they were excluded from the following analyses related to delayed allocentric navigation.

### Imaging Data Acquisition

All participants were scanned on a 3T MRI scanner with an 8-channel phased-array head coil (Philips, Achieva TX) at the Department of Radiology, Nanjing Drum Tower Hospital. The T_1_-weighted images (T_1_WI) were acquired with the following parameters: 192 sagittal slices, repetition time (TR) = 9.74 ms, echo time (TE) = 4.60 ms, slice thickness = 1 mm, field of view (FOV) = 256 × 256 mm^2^, and voxel size = 1 × 1 × 1 mm^3^.

### Basal Forebrain Subfield and Entorhinal Cortex Volumetry

MRI data were processed by the Computational Anatomy Toolbox (CAT12) for Statistics Parametric Mapping version 12 (SPM12). Briefly, MRI data were automatically segmented into gray matter (GM), white matter (WM), and cerebrospinal fluid (CSF) partitions. Then, the GM partitions were non-linearly normalized to the CAT12 default template (IXI555-MNI152) using the Diffeomorphic Anatomical Registration Through Exponentiated Lie Algebra (DARTEL) (Ashburner, 2007). Subject with a correlation between volumes that was two SDs below the mean suggested bad homogeneity of the data was excluded from the following analysis (Dahnke et al., 2013). The images were smoothed with a 4-mm full-width at half-maximum (FWHM) (Kilimann et al., 2014; Wolf et al., 2014). The GM, WM, and CSF partitions were summarized as the total intracranial volume (TIV), which was calculated to adjust for head size differences.

**Figure 2:**
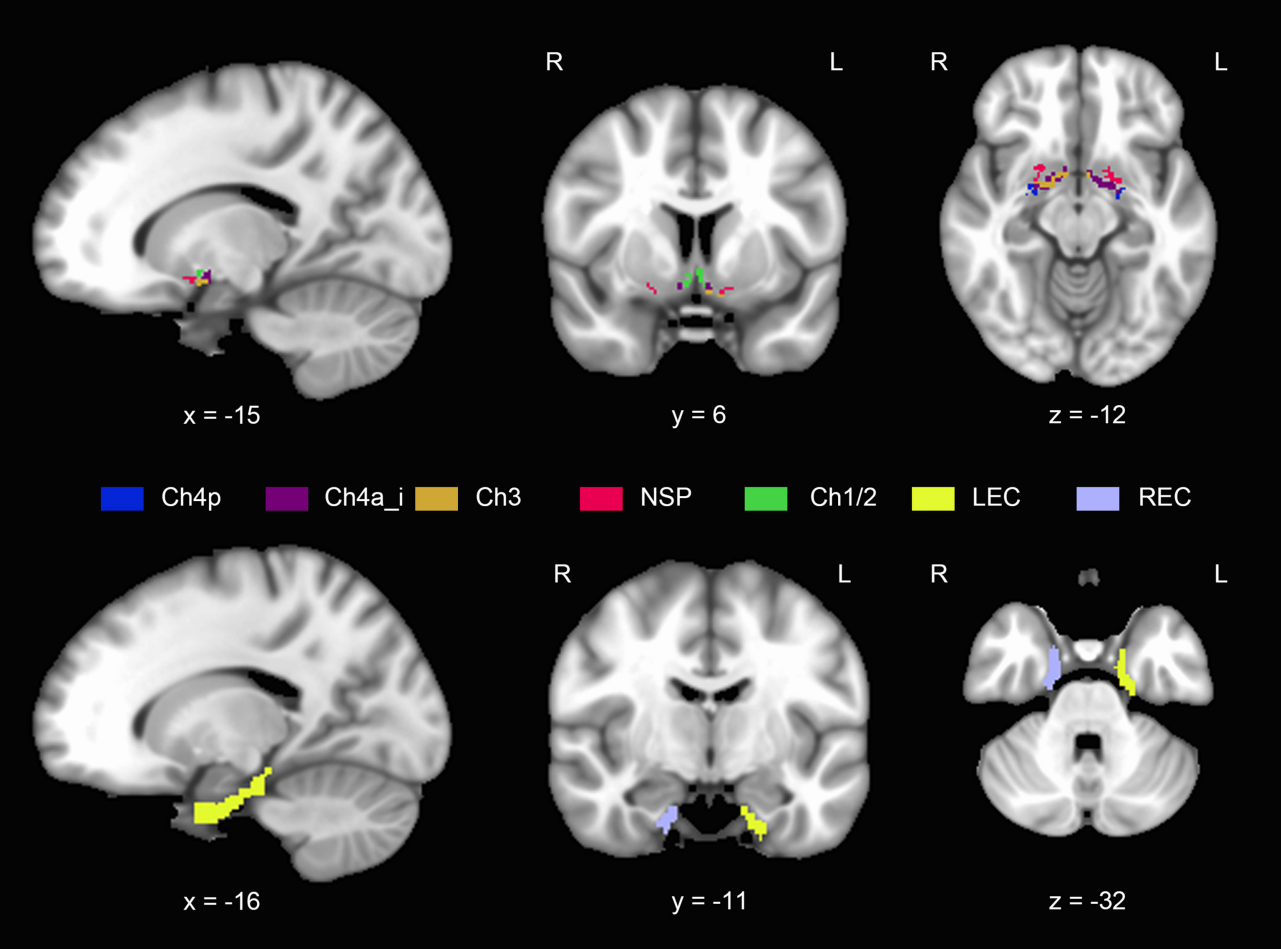
Anatomical position and extent of the basal forebrain and entorhinal cortex. Different colors refer to different subregions. NSP, nucleus subputaminalis; LEC, left entorhinal cortex; REC, right entorhinal cortex (For interpretation of the references to colors in this figure, the reader is referred to the web version of this article).

Calculation of the individual BF volumes was obtained by summing up the modulated GM voxel values within a cytoarchitectonic BF mask in the MNI space, which was derived from histological sections of a postmortem brain (Wolf et al., 2014). Regions of interest (ROIs) corresponding to the following BF subfields were derived (Figure 2): Ch1/2 (the nucleus of the vertical limb of the diagonal band), Ch3 (the nucleus of the horizontal limb of the diagonal band), Ch4a_i (anterior and intermediate parts of the nucleus basalis of Meynert), Ch4p (posterior part of the nucleus basalis of Meynert), and the nucleus subputaminalis (NSP). The entire volumes of the BF were defined as the sum of the volume of all subfields.

We extracted the subregions labeled 115 and 116 from the Brainnetome Atlas as the left EC and right EC mask, respectively (Fan et al., 2016) (Figure 2), using the Data Processing Assistant for Resting-State fMRI, advanced edition (DPARSF) (Chao-Gan and Yu-Feng, 2010). Individual EC volumes were calculated by summing up the modulated GM voxel values within the left or right EC mask.

We also calculated the hippocampal volume of each subject using FreeSurfer version 6.0.0 image analysis suites (http://freesurfer.net/), which was extracted as a covariate in subsequent correlation analyses.

### Apolipoprotein E Genotyping

DNA extraction from 300 μL of whole blood per subject was performed using an SK2884 DNA extraction kit (Sangon Biotech, Shanghai, China). Apolipoprotein E (APOE) single nucleotide polymorphism (SNP) genotyping was performed for rs429358 and rs7412 using PCR technology with the support of the BGI Tech Solutions Beijing Liuhe Company. We determined APOE ε4 status for 42 of the 55 participants (15/24 in the NC group and 27/31 in the SCD group).

### Statistical Analysis

Age, years of education, cognitive measures, and navigation distance errors were compared by two-sample *t*-tests. Gender distribution and APOE ε4 status were calculated by chi-square tests. We also applied paired t-tests to assess the differences in distance errors between egocentric and allocentric strategies within the whole cohort and in the NC and SCD cohorts. We also evaluated between-group differences in the total BF, BF subfields, EC, and hippocampal volumes, controlling for age, gender, years of education, and TIV.

The associations of SN errors with cognitive variables were assessed, adjusting for age, gender, and years of education. The correlations between the total BF, significant BF subfield volumes, total EC, and hippocampal volumes were calculated within the whole cohort and in the NC and SCD cohorts, adjusting for age, gender, years of education, and TIV. The associations between BF and EC volumetry and navigation distance errors on each subtest were also evaluated, adjusting for age, gender, years of education, TIV, and hippocampal volume. We further evaluated the differences in volumetry-navigation correlations between the two groups. Statistical analyses were performed with SPSS version 21.0 and the SurfStat package (http://www.math.mcgill.ca/keith/surfstat/). The significance level was set at *p* < 0.05 with two-tailed tests. Bonferroni corrections were applied for multiple comparisons.

## Results

### Demographic and Neuropsychological Data

As shown in Table 1, the SCD and NC groups did not significantly differ in age, gender distribution, or educational level. Following Bonferroni correction with an adjusted α of 0.003, the SCD group showed higher scores on the SCD-Q [*t*_(53)_ = −5.140, *p* < 0.001]. Under uncorrected criteria, the SCD group also performed worse on the ROCF recall test [*t*_(53)_ = 2.858, *p* = 0.006] and the SDMT [*t*_(53)_ = 2.034, *p* = 0.047]. No significant differences in MMSE scores, episodic memory, executive, language or visuospatial abilities were observed between the NC and SCD groups. The two groups did not significantly differ in APOE ε4 status [χ^2^_(1)_ = 0.380, *p* = 0.567].

**Table 1:** Demographic, neuropsychological, and APOE genotyping data.

	**NC (*n* = 24)**	**SCD (*n* = 31)**	**Statistics (degree of freedom)**	***P***
Age	63.50 ± 5.35	64.68 ± 5.21	*t*_(53)_ = −0.822	0.415
Gender	8/16	5/26	*χ^2^*_(1)_ = 2.218	0.136^a^
Education	13.25 ± 3.35	11.97 ± 2.60	*t*_(53)_ = 1.598	0.116
MMSE	29.04 ± 1.33	28.35 ± 1.43	*t*_(53)_ = 1.820	0.074
SCD-Q	3.19 ± 2.43	6.02 ± 1.65	*t*_(53)_ = −5.140	<0.001^**^
**Episodic memory**				
AVLT immediate	18.79 ± 4.75	16.55 ± 5.07	*t*_(53)_ = 1.673	0.100
AVLT short-term	5.83 ± 2.60	4.45 ± 2.57	*t*_(53)_ = 1.969	0.054
AVLT long-term	5.46 ± 2.50	4.39 ± 2.79	*t*_(53)_ = 1.477	0.146
AVLT cued recall	5.46 ± 2.17	4.19 ± 2.54	*t*_(53)_ = 1.953	0.056
AVLT recognition	21.67 ± 1.46	21.87 ± 1.15	*t*_(53)_ = −0.580	0.564
**Visuospatial memory**				
ROCF recall	18.13 ± 4.88	13.94 ± 5.76	*t*_(53)_ = 2.858	0.006^*^
**Executive function**				
TMT-A	58.04 ± 15.48	55.55 ± 17.34	*t*_(53)_ = 0.554	0.582
TMT-B	135.33 ± 46.04	153.32 ± 54.64	*t*_(53)_ = −1.295	0.201
**Language ability**				
AFT	19.17 ± 4.23	17.97 ± 4.57	*t*_(53)_ = 0.996	0.324
BNT	27.25 ± 2.71	27.16 ± 2.58	*t*_(53)_ = 0.124	0.902
**Visuospatial ability**				
ROCF copy	35.33 ± 1.24	34.23 ± 2.68	*t*_(53)_ = 1.873	0.067
CDT	27.67 ± 2.44	26.94 ± 2.71	*t*_(53)_ = 1.036	0.305
**Processing speed**				
SDMT	43.04 ± 9.68	37.45 ± 10.42	*t*_(53)_ = 2.034	0.047^*^
**Genotyping**				
APOE ε4 (carriers/non-carriers)	4/11	5/22	*χ^2^*_(1)_ = 0.380	0.537^a^^,^^b^

*Values are the mean ± SD*.

*NC, normal control; SCD, subjective cognitive decline; MMSE, Mini-Mental State Examination; SCD-Q, SCD questionnaire; AVLT, Auditory Verbal Learning Test; ROCF, Rey-Osterrieth Complex Figure; TMT-A, Trail Making Test part A; TMT-B, Trail Making Test part B; AFT, Animal Fluency Test; BNT, Boston Naming Test; CDT, Clock Drawing Test; SDMT, Symbol Digit Modalities Test; APOE, apolipoprotein E*.

* *p < 0.05, uncorrected*;

** *p < 0.003 (Bonferroni-adjusted α, 0.05/15 cognitive scales measured)*.

a *chi-square test*;

b *APOE ε4 status not determined for the whole cohort*.

### Comparisons of Navigation Behavior Performance and Associations With Cognitive Variables

As Table 2 and Figure 1 show, the SCD subjects demonstrated larger distance errors in all the navigation subtests than the controls [mixed alloegocentric navigation: *t*_(53)_ = −2.115, *p* = 0.039, Cohen's *d* = 0.60; egocentric navigation: *t*_(53)_ = −3.048, *p* = 0.004, Cohen's *d* = 0.88; allocentric navigation: *t*_(53)_ = −3.664, *p* < 0.001, Cohen's *d* = 1.03; delayed allocentric navigation: *t*_(51)_ = −3.328, *p* = 0.002, Cohen's *d* = 0.93], but the differences in mixed alloegocentric navigation errors did not survive Bonferroni correction with an adjusted α of 0.0125. In addition, the two groups did not significantly differ in average duration in each subtest.

**Table 2:** Spatial navigation distance errors.

	**NC (*n* = 24)**	**SCD (*n* = 31)**	**Statistics (degree of freedom)**	***P***	**Cohen's *d***
Mixed AEN	26.23 ± 9.86	35.60 ± 19.86	*t*_(53)_ = −2.115	0.039^*^	0.60
EN	20.63 ± 6.69	38.74 ± 28.44	*t*_(53)_ = −3.048	0.004^**^	0.88
AN	33.59 ± 15.74	57.35 ± 28.54	*t*_(53)_ = −3.664	<0.001^**^	1.03
DAN	28.16 ± 22.69	54.78 ± 33.27	*t*_(51)_ = −3.328	0.002^**^^,^^a^	0.93

*Average distance errors (in pixels) in mixed alloegocentric (AEN), egocentric (EN), allocentric (AN), and delayed allocentric navigation (DAN) subtests in the normal control (NC) and subjective cognitive decline (SCD) groups. Values are the mean ± SD*.

* *p < 0.05, uncorrected*;

** *p < 0.0125 (Bonferroni-adjusted α, 0.05/4 navigation tests measured)*.

a *Two SCD participants did not complete the DAN subtest; thus, they were excluded from the comparison*.

Regarding the within-group differences in two navigation strategies, we observed significantly larger distance errors in the allocentric strategy compared to the egocentric strategy in the whole [*t*_(54)_ = −5.519, *p* = < 0.001, Cohen's *d* = 0.74], NC [*t*_(23)_ = −4.458, *p* < 0.001, Cohen's *d* = 0.91], and SCD cohorts [*t*_(30)_ = −3.982, *p* < 0.001, Cohen's *d* = 0.72] (Supplementary Table 1).

Supplementary Figure 1 shows the correlations between SN errors and cognitive measures in the whole cohort adjusting for age, gender, and years of education. The ROCF recall scores showed significant negative associations with distance errors in all the SN subtests.

### Comparisons of BF, EC, and Hippocampal Volumes

**Figure 3:**
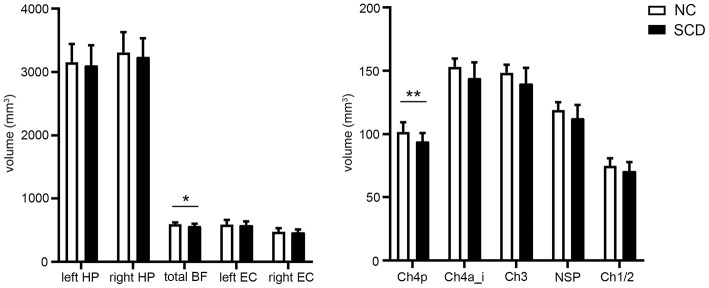
Group comparisons of the basal forebrain (BF), entorhinal cortex (EC), and hippocampal (HP) volumes between the normal control (NC) and subjective cognitive decline (SCD) groups. Values are the mean ± *SD*. ^*^p < 0.05; ^**^p < 0.01 (Bonferroni-adjusted α, 0.05/5 BF subfields measured). *P*-values were adjusted for age, gender, years of education, and total intracranial volume.

After adjusting for age, gender, education level, and TIV, the SCD group showed reduced total BF volumes compared to the NC group [*F*_(1)_ = 4.258, *p* = 0.044, partial η^2^ = 0.08] under uncorrected criteria. Considering the BF subfields, volume reduction in Ch4p in the SCD group [*F*_(1)_ = 8.187, *p* = 0.006, partial η^2^ = 0.14] survived the Bonferroni adjusted α of 0.01. No significant differences in total and bilateral EC volumes, and in total and bilateral hippocampal volumes were observed (Figure 3).

### Correlations Between BF Volumes and EC and Hippocampal Volumes

After adjusting for age, gender, education level, and TIV, we observed significant positive correlations between the Ch4p volumes and the total EC volumes (*r* = 0.332, *p* = 0.017) (Supplementary Figure 2A), and between total BF and hippocampal volumes (*r* = 0.369, *p* = 0.008) (Supplementary Figure 3A) in the whole cohort. In the SCD group, the total BF volumes showed positive correlations with total EC volumes (*r* = 0.394, *p* = 0.042) (Supplementary Figure 2B) and hippocampal volumes (*r* = 0.572, *p* = 0.002) (Supplementary Figure 3B). The Ch4p volumes also showed positive correlations with total EC volumes (*r* = 0.609, *p* < 0.001) (Supplementary Figure 2C) and hippocampal volumes (*r* = 0.558, *p* = 0.002) (Supplementary Figure 3C) in the SCD group. No significant associations between BF and EC volumes and between BF and hippocampal volumes were observed in the NC group (Supplementary Table 2).

### Correlations Between BF and EC Volumes and Navigation Performance

**Figure 4:**
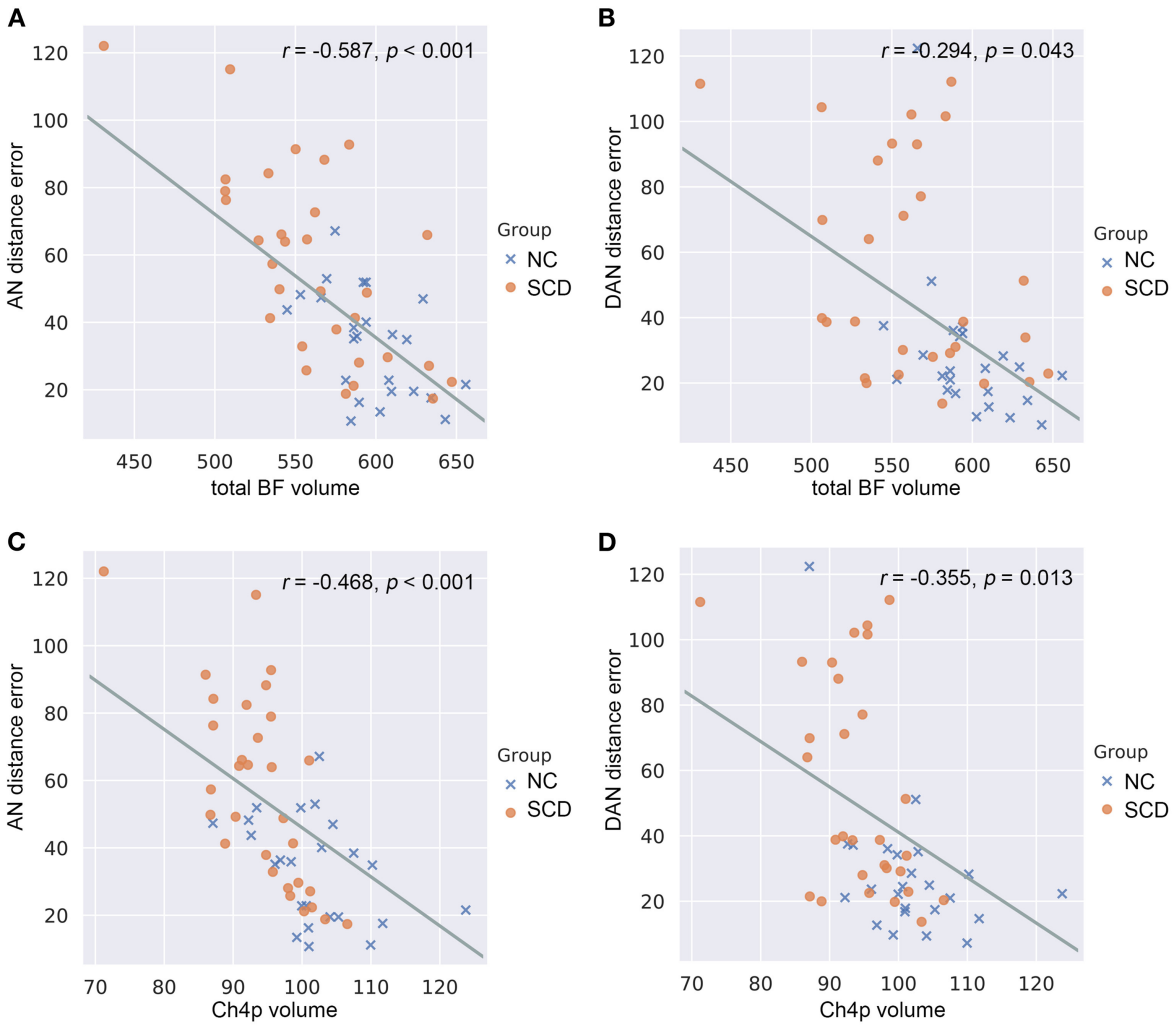
Correlations between basal forebrain (BF) volumetry and navigation distance errors in the whole cohort. **(A)** Correlations between allocentric navigation (AN) distance errors and total BF volumes. **(B)** Correlations between delayed allocentric navigation (DAN) distance errors and total BF volumes. **(C)** Correlations between AN distance errors and Ch4p volumes. **(D)** Correlations between DAN distance errors and Ch4p volumes. NC, normal control; SCD, subjective cognitive decline. *P*-values were adjusted for age, gender, years of education, total intracranial volume, and hippocampal volume.

In the whole cohort (Supplementary Table 3), the total BF volumes were negatively correlated with allocentric errors (*r* = −0.587, *p* < 0.001) (Figure 4A) and delayed allocentric errors (*r* = −0.294, *p* = 0.043) (Figure 4B). The Ch4p volumes were negatively correlated with both allocentric errors (*r* = −0.468, *p* < 0.001) (Figure 4C) and delayed allocentric errors (*r* = −0.355, *p* = 0.013) (Figure 4D), controlling for age, gender, years of education, TIV, and hippocampal volume.

**Figure 5:**
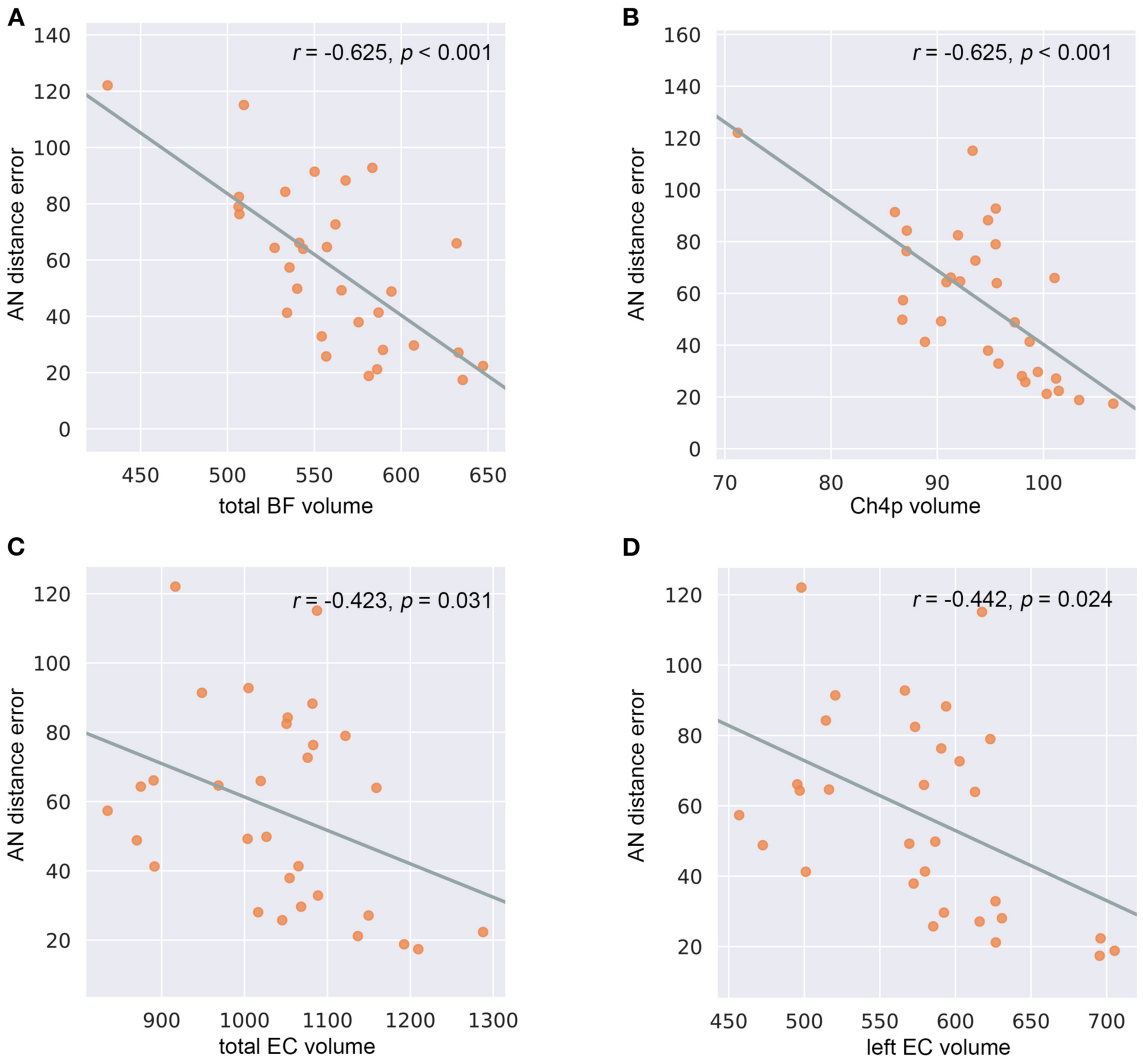
Correlations between basal forebrain (BF) and entorhinal cortex (EC) volumetry and navigation distance errors in the subjective cognitive decline (SCD) cohort. **(A)** Correlations between allocentric navigation (AN) distance errors and total BF volumes. **(B)** Correlations between AN distance errors and Ch4p volumes. **(C)** Correlations between AN distance errors and total EC volumes. **(D)** Correlations between AN distance errors and left EC volumes. *P*-values were adjusted for age, gender, years of education, total intracranial volume, and hippocampal volume.

In the SCD group (Supplementary Table 4), the reduced total BF volumes were associated with larger allocentric errors (*r* = −0.625, *p* < 0.001) (Figure 5A), and the reduced Ch4p volumes were associated with larger allocentric errors (*r* = −0.625, *p* < 0.001) (Figure 5B). We also observed a negative correlation between the total EC volumes and allocentric errors (*r* = −0.423, *p* = 0.031) (Figure 5C) and between the left EC volumes and allocentric errors (*r* = −0.442, *p* = 0.024) (Figure 5D) in the SCD group, controlling for age, gender, years of education, TIV, and hippocampal volume.

In the NC group (Supplementary Table 5), no significant associations between BF, EC volumes, and navigation errors were observed.

### Comparisons of Correlations Between BF and EC Volumes and Navigation Performance

Regarding the brain-behavior correlation comparison, we observed significant differences in the Ch4p volume-allocentric error correlation [*F*_(1, 46)_ = 10.07, *p* = 0.003], total EC volume-allocentric error correlation [*F*_(1, 46)_ = 4.75, *p* = 0.034], and left EC volume-allocentric error correlation [*F*_(1, 46)_ = 5.22, *p* = 0.027] between the NC and SCD groups, controlling for age, gender, years of education, TIV, and hippocampal volume (Supplementary Figure 4).

## Discussion

In the present study, we observed worse egocentric and allocentric navigation performance in the SCD subjects. Additionally, we found that SCD subjects showed reduced volumes in the Ch4p subfield of BF, which were negatively correlated with allocentric distance errors. Our findings support the hypothesis that BF atrophy and spatial disorientation are objective and sensitive biomarkers for the preclinical detection of subjects with potential AD and point to the critical role of the BF, especially the Ch4p subfield, in allocentric disorientation in the SCD stage.

### SCD Subjects Showed Egocentric and Allocentric Disorientation

With the exception of SCD-Q, the SCD subjects revealed comparable function to the controls in all the cognitive domains based on the neuropsychological evaluation after Bonferroni correction. Regarding the navigation test, the SCD subjects showed disorientation with both egocentric and allocentric representations, consistent with that observed in MCI and AD dementia patients in previous studies using the same paradigm (Hort et al., 2007; Laczo et al., 2010). Notably, the study by Hort et al. (2007) did not reveal significant differences in navigation performance between NCs and participants with subjective memory complaints (SMC). We speculated that differences in diagnostic criteria for SMC and SCD, sample size, and demographic characteristics may be possible factors contributing to the discrepancies. Our study extended previous findings by showing that spatial deficits exist in preclinical subjects at a higher risk of AD.

Egocentric is self-centered and depends on the parietal cortex and caudate nucleus, while allocentric is world-centered and hippocampus-driven (Laczó et al., 2018). Navigation likely represents a distinguishable cognitive domain that could provide promising methods for detecting individuals with incipient AD. In the present study, navigation performance revealed significant correlations with a broad range of cognitive domains, especially visuospatial memory, visuospatial ability, and processing speed. These relationships support the notion that SN is a complex process that associates with various navigational skills including spatial memory and visuospatial ability (Botly and De Rosa, 2009; Lithfous et al., 2013; Li and King, 2019). A recent study has also suggested a relation between SN impairments and processing speed (Glikmann-Johnston et al., 2019). Of note, compared to the traditional cognitive scales, navigation tests could overcome limits based on ethnic origins and cultural restrictions, which may benefit longitudinal studies with large cohorts in the future (Coughlan et al., 2018).

Regarding the within-group analysis, we found that participants showed larger distance errors in allocentric compared to egocentric navigation. According to previous studies, older people prefer to use the egocentric strategy for navigation (Harris et al., 2012; Wiener et al., 2013; Lester et al., 2017). Our findings suggest that more accurate navigation using the egocentric rather than allocentric strategy may be an explanation for this bias. Still, this might also imply the absence of significant associations between mixed alloegocentric distance errors and BF volumes while the presence of strong correlations between allocentric performance and BF volumes discussed below in the present study, considering old subjects might tend to choose egocentric strategy when both egocentric and allocentric references were provided.

### SCD Subjects Showed Reduced Ch4p Subfield Volumes of BF

We observed reduced Ch4p subfield volumes of BF in the SCD group compared to the NC group. Postmortem studies have documented cholinergic neuron loss in the BF in AD patients (Vogels et al., 1990), which was most pronounced in the Ch4p region (Liu et al., 2015). Previous studies have demonstrated significant volume reductions in all BF subfields except for Ch2 in MCI patients in a multicentre cohort, and the subsequent receiver operating characteristics (ROC) analysis for the separation between subjects with MCI and NCs revealed a higher diagnostic value of the Ch4p region than the hippocampus (Kilimann et al., 2014). A recent study investigating BF volumes in SCD subjects showed a significant total volume reduction in the BF, with the largest effect sizes in the Ch1/2 and Ch4p subregions, and the latter was associated with reduced glucose metabolism in the right precuneus, which had been reported to predict subsequent memory decline (Scheef et al., 2019). In addition, studies have reported negative correlations between BF volume and cortical amyloid deposition in presymptomatic subjects, suggesting intrinsic associations between cholinergic degeneration and amyloid pathology in the preclinical stages of AD (Grothe et al., 2014). Our findings provide evidence that SCD represents a higher risk of preclinical AD from the perspective of BF volumetry, which also suggests that Ch4p atrophy may serve as a sensitive imaging marker for the identification of incipient AD patients.

By contrast, we did not find significant differences in EC and hippocampal volumes between the two groups, which has been considered the earliest regions demonstrating neurofibrillary tangles and amyloid deposition in the initial stages of AD (Braak and Del Tredici, 2015). Previous studies have shown cortical thinning or reduced volumes of EC and hippocampus in SCD subjects, which reflected early alterations related to AD pathology in the SCD stage (Jessen et al., 2006; Saykin et al., 2006; Meiberth et al., 2015; Ryu et al., 2017; Zhao et al., 2019). Similar to our findings, these studies also did not find significant EC or hippocampal volumetry differences between controls and SCD subjects (Selnes et al., 2012; Hong et al., 2016; Ryu et al., 2017). Factors such as SCD definition, recruitment site, and calculation methods may contribute to the discrepant results. Notably, the SCD participants in the present study may be in a relatively earlier phase of SCD, while the SCD cohorts in previous studies showing remarkable EC or hippocampal atrophy may be representative of a later phase of SCD that is closer to MCI.

Since the SCD cohort in the present study showed volume reductions in the Ch4p subfield of BF while comparable EC and hippocampal volumes with the controls, we speculated that reduced BF volumes might have an advantage over EC or hippocampal atrophy as sensitive imaging markers for the detection of potential AD patients. However, since no pathology biomarkers and no follow-up data were available, the conclusion that cholinergic degeneration of the BF precedes neurofibrillary tangles or amyloid deposits in the EC and hippocampus in the initial stage of AD should be made with caution. In line with previous studies (Kerbler et al., 2015a,b)(Kerbler et al., 2015a,b), the positive relationships between BF and EC and hippocampal volumetry observed in the whole cohort and the SCD group may suggest covariation of these pathological processes, which remains to be further validated by studies with AD pathology biomarkers and more accurate volumetric methods.

### BF Atrophy, Especially in the Ch4p Subfield, Contributed to Allocentric Disorientation in SCD Subjects

In the whole cohort, greater BF and Ch4p volumes were associated with better allocentric navigation performance. Studies in rats have revealed the role of cholinergic neurons in the posterior BF in visuospatial attention during feature binding (Botly and De Rosa, 2012). Furthermore, cholinesterase inhibitors have been reported to increase the selectivity of neural responses during visual working memory encoding in humans, which are crucial for allocentric navigation (Furey et al., 2000). Our findings were consistent with previous studies in that greater BF volumes predicted better allocentric navigation ability.

In the SCD group, the significant correlations between total BF and Ch4p volumes and allocentric errors suggested that BF degeneration, especially in the Ch4p subfield, contributes to allocentric disorientation in SCD subjects. Previous studies have demonstrated marked correlations between allocentric performance and anterior BF volumes, which covered Ch1-3 and the anterior region of Ch4, while no significant correlations between egocentric performance and BF volumes were found in AD dementia patients (Kerbler et al., 2015b). However, we did not observe significant Ch1-2 and Ch3 volume reductions in the SCD group, indicating that the allocentric disorientation may not be due to Ch1-3 atrophy in the preclinical stage. Previous studies also suggested that AD-related neurodegenerative changes in the BF may lead to less effective allocentric processing and increased reliance on egocentric representations in the early clinical stages of AD (Parizkova et al., 2018). Furthermore, mild AD patients treated with cholinesterase inhibitors demonstrated improved delayed allocentric performance after 3 months (Hort et al., 2014). Our findings provide additional evidence that Ch4p atrophy contributes to allocentric navigation deficits in the SCD stage independent of hippocampal atrophy and have implications for the potential use of the SN test for prognostic evaluation of drugs targeting the cholinergic system in preclinical AD patients.

The Ch4 region mainly projects to the medial frontal, cingulate, retrosplenial, and visual cortices (Solari and Hangya, 2018). The medial frontal cortex has been implicated in the upstream processing of spatial memory (Ito, 2018). The retrosplenial cortex has been identified as crucial for allocentric navigation and the flexible transition between egocentric representations and allocentric representations (Vann et al., 2009). Ch4p atrophy may lead to disrupted projections from the BF to the medial frontal cortex and retrosplenial cortex and thus subsequent allocentric deficits. Although Ch4 also projects to the posterior parietal cortex (PPC), which mainly contributes to egocentric route planning, we did not observe significant correlations between Ch4p volumes and egocentric performance. We speculated that the Ch4-PPC cholinergic neurons were insusceptible to the earliest AD-related alterations and thus did not predict egocentric deficits in the SCD stage. Longitudinal studies with direct detection of functional assessment of cholinergic activity rather than mere BF volumetry are needed to further elucidate these speculations.

Neurons in Ch4p also project to the adjacent EC (Mesulam et al., 1983; Parizkova et al., 2018). We observed marked associations between total and left EC volumes and allocentric performance in the SCD group. The EC, particularly the medial part, processes self-motion generated and environmental landmark orienting signals to create an allocentric representation (Wang et al., 2020). In addition, the medial EC contains grid cells, which encode spatial information to form a cognitive map critical for allocentric strategies. Critically, young adults at genetic risk of AD (APOE ε4 carriers) exhibited reduced grid-cell-like representations and altered SN behavior in a virtual arena (Kunz et al., 2015). No significant associations between EC volumetry and navigation performance were detected either in the whole cohort or in the NC group. Therefore, we speculated that the negative relationships between EC volumes and allocentric errors did not represent a normal aging process but an SCD-related covariation.

The SCD vs. NC group difference may also modulate the relationship between BF and EC volumes and navigation behavior, with greater volume predicting better performance being more evident in the SCD group. These findings highlighted that stage specificity should be taken into consideration while investigating the associations between brain measures and behavior in AD-related studies (Qing et al., 2017).

## Limitations

This study has some limitations. First, we conducted this cross-sectional study in a small cohort, which was mainly composed of female subjects; thus, enlarging the sample size, increasing the number of male participants, and collecting follow-up data is necessary for our future studies. Second, the SN test was performed on the computer, which might be difficult for participants with no computer experience, although the skill demands were relatively basic. Notably, although the computerized SN test has been suggested highly associated with the real-space SN test (Hort et al., 2007), we need to examine SN ability in virtual reality or real space in our future study to make the present findings more convincing (Coughlan et al., 2018). Third, since preclinical AD is a designation for individuals who exhibit pathological amyloid-β and tau deposits, it is critical to collect data on these biomarkers and direct evidence of cholinergic neurodegeneration in our future research, which may benefit a better understanding of the directionality between reports of SCD and BF atrophy. Further, a recent study has reported the effects of APOE ε4 on navigation (Coughlan et al., 2020), thus in our future study with a larger sample size, we need to regress out the potential effects of APOE genotype. Last, a more sophisticated EC mask containing subregions is needed, since the posteromedial part of the EC was believed to be more relevant to SN than the anterolateral part (Howett et al., 2019). Longitudinal studies with large cohorts, novel navigation paradigms, and sophisticated segmentation methods are needed for the systemic clarification of the neural basis underlying spatial deficits in SCD individuals in the future.

## Conclusion

In the present study, we observed spatial disorientation in the SCD subjects, which may serve as a promising biomarker for the early detection of potential AD patients and indicate future cognitive deterioration. Furthermore, the volume reductions in the Ch4p subfield of BF suggested the structural neural basis for allocentric navigation deficits in the SCD stage. Our findings may provide novel insights into the early diagnosis and prognostic evaluation of subjects at higher risk of incipient AD.

## Data Availability Statement

The raw data supporting the conclusions of this article will be made available by the authors, without undue reservation.

## Ethics Statement

The studies involving human participants were reviewed and approved by Human Participants Ethics Committee of Nanjing Drum Tower Hospital. The patients/participants provided their written informed consent to participate in this study.

## Author Contributions

QC was responsible for the conception and design of the present study, execution of the experimental work, and wrote the first draft of the manuscript. SW and XL undertook the conception of the study and the review and critique of the manuscript. YS and WC executed the experimental work. JLu, WZ, JLi, and ZQ organized the research project and reviewed and critiqued the statistical analysis. ZN and JH provided the computerized spatial navigation test paradigm. XZ and BZ guided the design of the study protocol and reviewed and critiqued the manuscript. All authors contributed to the article and approved the submitted version.

## Conflict of Interest

The authors declare that the research was conducted in the absence of any commercial or financial relationships that could be construed as a potential conflict of interest.

## Supplementary Material

Supplementary Figure 1 Correlations between spatial navigation distance errors and cognitive variables in the whole cohort. AEN, mixed alloegocentric navigation; EN, egocentric navigation; AN, allocentric navigation; DAN, delayed allocentric navigation; MMSE, Mini-mental state examination; SCD-Q, subjective cognitive decline questionnaire; AVLT, auditory verbal learning test; ROCF, Rey-Osterrieth complex figure; TMT-A, trail making test part A; TMT-B, trail making test part B; AFT, animal fluency test; BNT, Boston naming test; CDT, clock drawing test; SDMT, symbol digit modalities test. ^*^*p* < 0.05; ^**^*p* < 0.01; ^***^*p* < 0.001. Findings were adjusted for age, gender, and years of education.

Supplementary Figure 2 Correlations between basal forebrain (BF) and entorhinal cortex (EC) volumes. NC, normal control; SCD, subjective cognitive decline. *P*-values were adjusted for age, gender, years of education, and total intracranial volume.

Supplementary Figure 3 Correlations between basal forebrain (BF) and hippocampal (HP) volumes. NC, normal control; SCD, subjective cognitive decline. *P*-values were adjusted for age, gender, years of education, and total intracranial volume.

Supplementary Figure 4 Comparisons of the correlations between basal forebrain (BF) and entorhinal cortex (EC) volumetry and spatial navigation distance errors between the subjective cognitive decline (SCD) and normal control (NC) groups. AN, allocentric navigation. *P*-values were adjusted for age, gender, years of education, total intracranial volume, and hippocampal volume.

Supplementary Table 1 Comparisons of navigation distance errors between egocentric and allocentric strategies.Average distance errors (in pixels) in egocentric navigation (EN) and allocentric navigation (AN) subtests within the whole cohort, normal control (NC), and subjective cognitive decline (SCD) groups. Values are the mean ± SD. ^*^*p* < 0.05.

Supplementary Table 2 Correlations between total EC and HP volumes and BF volumes.BF, basal forebrain; EC, entorhinal cortex; HP, hippocampus; NC, normal control; SCD, subjective cognitive decline. ^*^*p* < 0.05. P values were adjusted for age, gender, years of education, and total intracranial volume.

Supplementary Table 3 Correlations between BF and EC volumetry and navigation distance errors in the whole cohort.AEN, alloegocentric navigation; EN, egocentric navigation; AN, allocentric navigation; DAN, delayed allocentric navigation; BF, basal forebrain; EC, entorhinal cortex. ^*^*p* < 0.05. P values were adjusted for age, gender, years of education, total intracranial volume, and hippocampal volume.

Supplementary Table 4 Correlations between BF and EC volumetry and navigation distance errors in the SCD group.SCD, subjective cognitive decline; AEN, alloegocentric navigation; EN, egocentric navigation; AN, allocentric navigation; DAN, delayed allocentric navigation; BF, basal forebrain; EC, entorhinal cortex. ^*^*p* < 0.05. P values were adjusted for age, gender, years of education, total intracranial volume, and hippocampal volume.

Supplementary Table 5 Correlations between BF and EC volumetry and navigation distance errors in the NC group. NC, normal control; AEN, alloegocentric navigation; EN, egocentric navigation; AN, allocentric navigation; DAN, delayed allocentric navigation; BF, basal forebrain; EC, entorhinal cortex. P values were adjusted for age, gender, years of education, total intracranial volume, and hippocampal volume.

Supplementary Box 1 Subjective cognitive decline questionnaire.
