# Supplementary figures and images for: Basal Forebrain Atrophy Is Associated With Allocentric Navigation Deficits in Subjective Cognitive Decline
Source: Front Aging Neurosci. 2021 Feb 15;13:596025. doi: 10.3389/fnagi.2021.596025 (PMC7917187; doi:10.3389/fnagi.2021.596025)

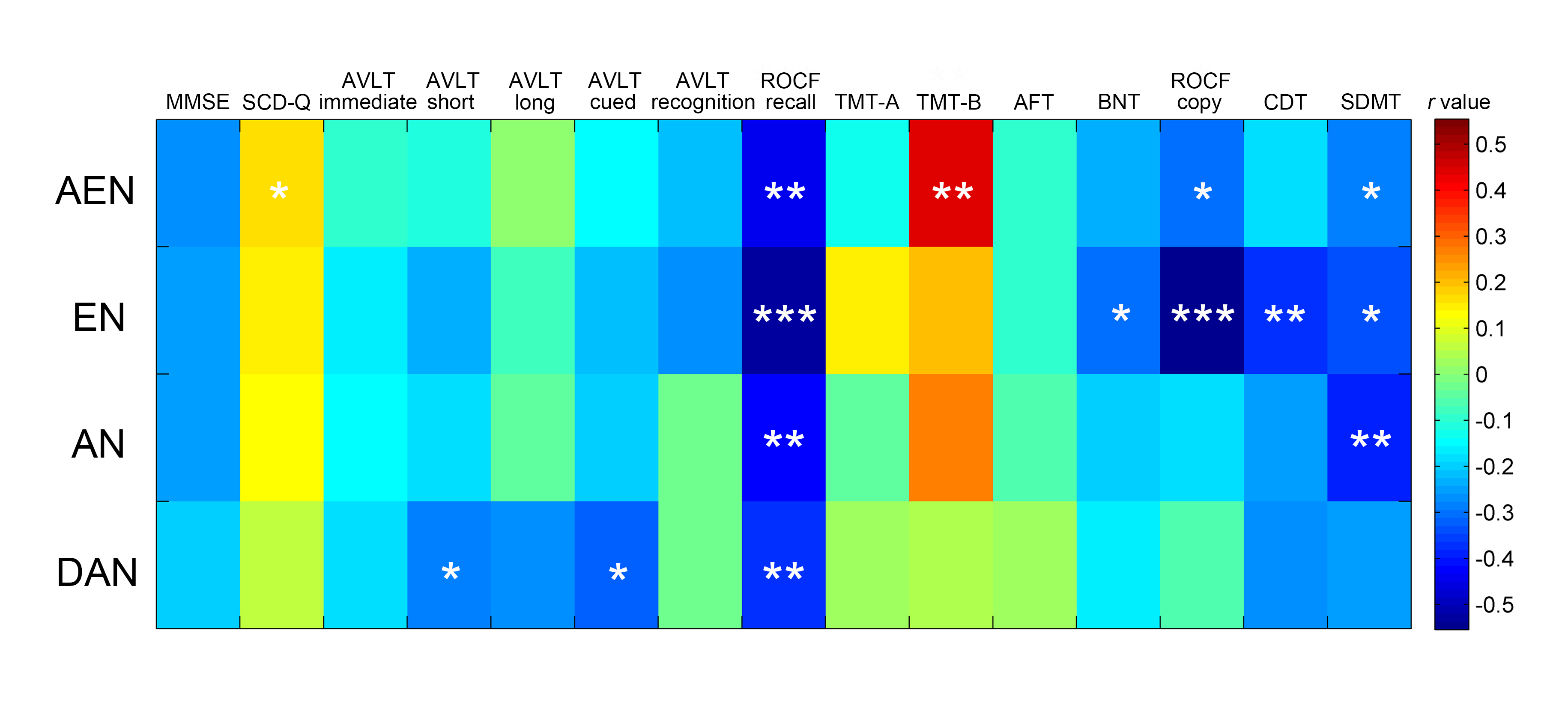

Supplement: Supplementary Figure 1 — Correlations between spatial navigation distance errors and cognitive variables in the whole cohort. AEN, mixed alloegocentric navigation; EN, egocentric navigation; AN, allocentric navigation; DAN, delayed allocentric navigation; MMSE, Mini-mental state examination; SCD-Q, subjective cognitive decline questionnaire; AVLT, auditory verbal learning test; ROCF, Rey-Osterrieth complex figure; TMT-A, trail making test part A; TMT-B, trail making test part B; AFT, animal fluency test; BNT, Boston naming test; CDT, clock drawing test; SDMT, symbol digit modalities test. *p < 0.05; **p < 0.01; ***p < 0.001. Findings were adjusted for age, gender, and years of education. [file Image_1.TIF]

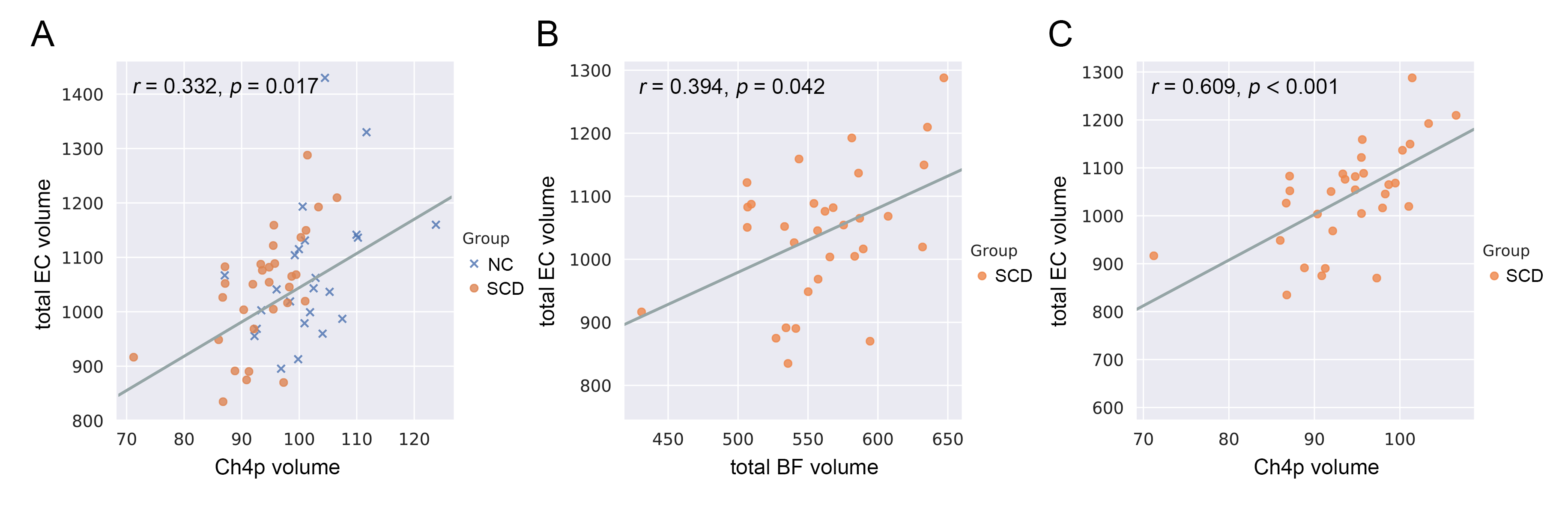

Supplement: Supplementary Figure 2 — Correlations between basal forebrain (BF) and entorhinal cortex (EC) volumes. NC, normal control; SCD, subjective cognitive decline. P-values were adjusted for age, gender, years of education, and total intracranial volume. [file Image_2.TIF]

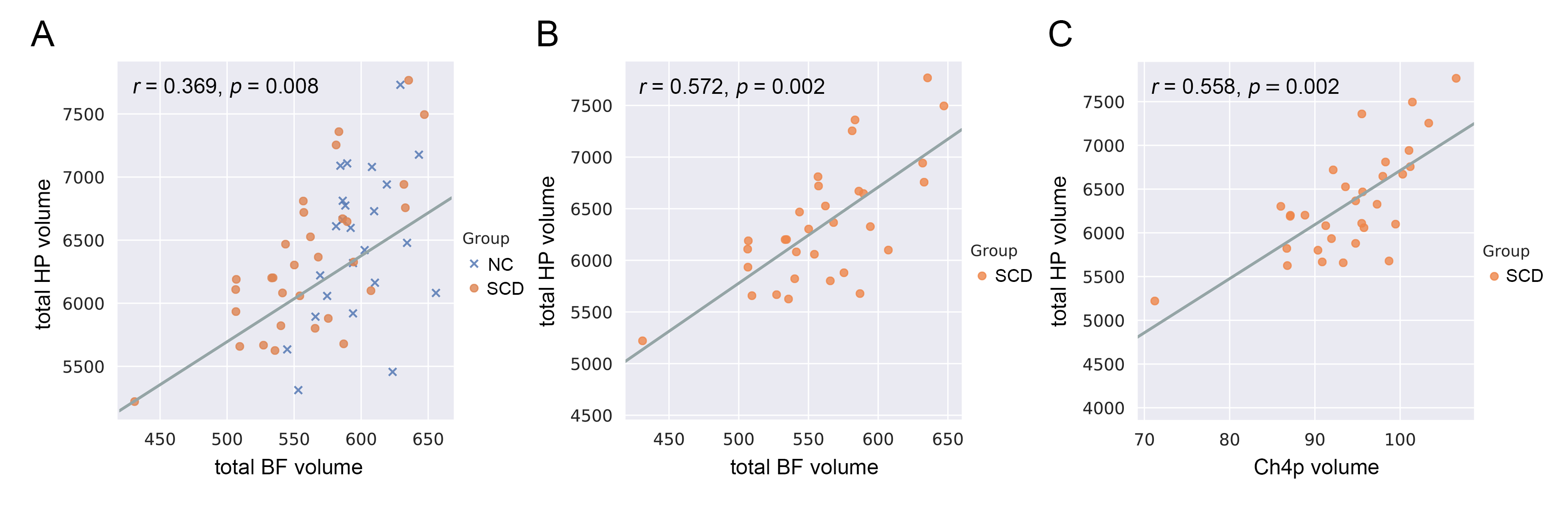

Supplement: Supplementary Figure 3 — Correlations between basal forebrain (BF) and hippocampal (HP) volumes. NC, normal control; SCD, subjective cognitive decline. P-values were adjusted for age, gender, years of education, and total intracranial volume. [file Image_3.TIF]

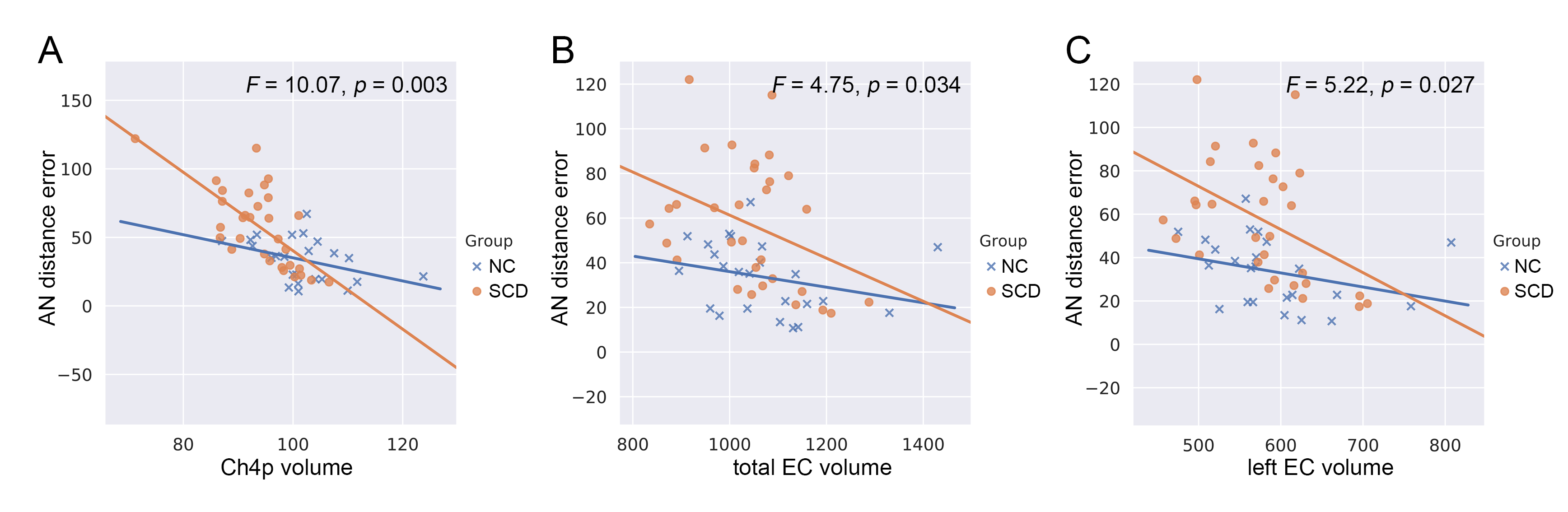

Supplement: Supplementary Figure 4 — Comparisons of the correlations between basal forebrain (BF) and entorhinal cortex (EC) volumetry and spatial navigation distance errors between the subjective cognitive decline (SCD) and normal control (NC) groups. AN, allocentric navigation. P-values were adjusted for age, gender, years of education, total intracranial volume, and hippocampal volume. [file Image_4.TIF]
