# Supplementary material for: Basal Forebrain Atrophy Is Associated With Allocentric Navigation Deficits in Subjective Cognitive Decline
Source: Front Aging Neurosci. 2021 Feb 15;13:596025. doi: 10.3389/fnagi.2021.596025 (PMC7917187; doi:10.3389/fnagi.2021.596025)
Supplement: Supplementary Table 1 — Comparisons of navigation distance errors between egocentric and allocentric strategies.Average distance errors (in pixels) in egocentric navigation (EN) and allocentric navigation (AN) subtests within the whole cohort, normal control (NC), and subjective cognitive decline (SCD) groups. Values are the mean ± SD. *p < 0.05. [file Table_1.docx]

Supplementary Table 1 Comparisons of navigation distance errors between egocentric and allocentric strategies.

|  | EN | AN | *t* | *p* | Cohen’s d |
| --- | --- | --- | --- | --- | --- |
| Whole cohort | 30.84±23.46 | 46.98±26.45 | *t*_(54)_ = -5.519 | <0.001* | 0.74 |
| NC cohort | 20.63±6.69 | 33.59±15.74 | *t*_(23)_ = -4.458 | <0.001* | 0.91 |
| SCD cohort | 38.74±28.44 | 57.35±28.54 | *t*_(30)_ = -3.982 | <0.001* | 0.72 |

Average distance errors (in pixels) in egocentric navigation (EN) and allocentric navigation (AN) subtests within the whole cohort, normal control (NC), and subjective cognitive decline (SCD) groups. Values are the mean ± SD. *: *p* < 0.05.
