# Supplementary material for: Basal Forebrain Atrophy Is Associated With Allocentric Navigation Deficits in Subjective Cognitive Decline
Source: Front Aging Neurosci. 2021 Feb 15;13:596025. doi: 10.3389/fnagi.2021.596025 (PMC7917187; doi:10.3389/fnagi.2021.596025)
Supplement: Supplementary Table 2 — Correlations between total EC and HP volumes and BF volumes.BF, basal forebrain; EC, entorhinal cortex; HP, hippocampus; NC, normal control; SCD, subjective cognitive decline. *p < 0.05. P values were adjusted for age, gender, years of education, and total intracranial volume. [file Table_2.docx]

Supplementary Table 2 Correlations between total EC and HP volumes and BF volumes.

|  | | Whole cohort | | NC cohort | | SCD cohort | |
| --- | --- | --- | --- | --- | --- | --- | --- |
|  |  | total EC | total HP | total EC | total HP | total EC | total HP |
| total BF | *r* | 0.197 | 0.369 | 0.099 | 0.129 | 0.394 | 0.572 |
|  | *p* | 0.165 | 0.008* | 0.678 | 0.587 | 0.042* | 0.002* |
| Ch4p | *r* | 0.332 | 0.202 | 0.043 | -0.091 | 0.609 | 0.558 |
|  | *p* | 0.017* | 0.154 | 0.857 | 0.702 | <0.001* | 0.002* |

BF: basal forebrain; EC: entorhinal cortex; HP: hippocampus; NC: normal control; SCD: subjective cognitive decline. *: *p* < 0.05. *P* values were adjusted for age, gender, years of education, and total intracranial volume.
