# Supplementary material for: Basal Forebrain Atrophy Is Associated With Allocentric Navigation Deficits in Subjective Cognitive Decline
Source: Front Aging Neurosci. 2021 Feb 15;13:596025. doi: 10.3389/fnagi.2021.596025 (PMC7917187; doi:10.3389/fnagi.2021.596025)
Supplement: Supplementary Table 3 — Correlations between BF and EC volumetry and navigation distance errors in the whole cohort.AEN, alloegocentric navigation; EN, egocentric navigation; AN, allocentric navigation; DAN, delayed allocentric navigation; BF, basal forebrain; EC, entorhinal cortex. *p < 0.05. P values were adjusted for age, gender, years of education, total intracranial volume, and hippocampal volume. [file Table_3.docx]

Supplementary Table 3 Correlations between BF and EC volumetry and navigation distance errors in the whole cohort.

|  | Whole cohort | | | | | | | | | | |
| --- | --- | --- | --- | --- | --- | --- | --- | --- | --- | --- | --- |
|  | total BF | | Ch4p | | total EC | | left EC | | right EC | | |
|  | *r* | *p* | *r* | *p* | *r* | *p* | *r* | *p* | | *r* | *p* |
| Mixed AEN | -0.018 | 0.900 | -0.137 | 0.342 | 0.011 | 0.941 | 0.013 | 0.926 | | 0.005 | 0.972 |
| EN | -0.274 | 0.054 | -0.076 | 0.599 | 0.097 | 0.504 | 0.115 | 0.428 | | 0.054 | 0.708 |
| AN | -0.587 | <0.001* | -0.468 | <0.001* | -0.136 | 0.346 | -0.177 | 0.220 | | -0.059 | 0.684 |
| DAN | -0.294 | 0.043* | -0.355 | 0.013* | -0.010 | 0.947 | -0.001 | 0.996 | | -0.018 | 0.905 |

AEN: alloegocentric navigation; EN: egocentric navigation; AN: allocentric navigation; DAN: delayed allocentric navigation; BF: basal forebrain; EC: entorhinal cortex. *: *p* < 0.05. *P* values were adjusted for age, gender, years of education, total intracranial volume, and hippocampal volume.
