# Supplementary material for: Basal Forebrain Atrophy Is Associated With Allocentric Navigation Deficits in Subjective Cognitive Decline
Source: Front Aging Neurosci. 2021 Feb 15;13:596025. doi: 10.3389/fnagi.2021.596025 (PMC7917187; doi:10.3389/fnagi.2021.596025)
Supplement: Supplementary Table 4 — Correlations between BF and EC volumetry and navigation distance errors in the SCD group.SCD, subjective cognitive decline; AEN, alloegocentric navigation; EN, egocentric navigation; AN, allocentric navigation; DAN, delayed allocentric navigation; BF, basal forebrain; EC, entorhinal cortex. *p < 0.05. P values were adjusted for age, gender, years of education, total intracranial volume, and hippocampal volume. [file Table_4.docx]

Supplementary Table 4 Correlations between BF and EC volumetry and navigation distance errors in the SCD group.

|  | SCD cohort | | | | | | | | | | |
| --- | --- | --- | --- | --- | --- | --- | --- | --- | --- | --- | --- |
|  | total BF | | Ch4p | | total EC | | left EC | | right EC | | |
|  | *r* | *p* | *r* | *p* | *r* | *p* | *r* | *p* | | *r* | *p* |
| Mixed AEN | 0.062 | 0.763 | -0.154 | 0.452 | -0.211 | 0.301 | -0.238 | 0.242 | | -0.130 | 0.526 |
| EN | -0.316 | 0.116 | -0.033 | 0.874 | 0.022 | 0.917 | -0.019 | 0.925 | | 0.067 | 0.744 |
| AN | -0.625 | <0.001* | -0.625 | <0.001* | -0.423 | 0.031* | -0.442 | 0.024* | | -0.305 | 0.129 |
| DAN | -0.227 | 0.287 | -0.370 | 0.075 | -0.169 | 0.430 | -0.139 | 0.516 | | -0.170 | 0.427 |

SCD: subjective cognitive decline; AEN: alloegocentric navigation; EN: egocentric navigation; AN: allocentric navigation; DAN: delayed allocentric navigation; BF: basal forebrain; EC: entorhinal cortex. *: *p* < 0.05. *P* values were adjusted for age, gender, years of education, total intracranial volume, and hippocampal volume.
