# Supplementary material for: Basal Forebrain Atrophy Is Associated With Allocentric Navigation Deficits in Subjective Cognitive Decline
Source: Front Aging Neurosci. 2021 Feb 15;13:596025. doi: 10.3389/fnagi.2021.596025 (PMC7917187; doi:10.3389/fnagi.2021.596025)
Supplement: Supplementary Table 5 — Correlations between BF and EC volumetry and navigation distance errors in the NC group. NC, normal control; AEN, alloegocentric navigation; EN, egocentric navigation; AN, allocentric navigation; DAN, delayed allocentric navigation; BF, basal forebrain; EC, entorhinal cortex. P values were adjusted for age, gender, years of education, total intracranial volume, and hippocampal volume. [file Table_5.docx]

Supplementary Table 5 Correlations between BF and EC volumetry and navigation distance errors in the NC group.

|  | NC cohort | | | | | | | | | |
| --- | --- | --- | --- | --- | --- | --- | --- | --- | --- | --- |
|  | total BF | | Ch4p | | total EC | | left EC | | right EC | |
|  | *r* | *p* | *r* | *p* | *r* | *p* | *r* | *p* | *r* | *p* |
| Mixed AEN | -0.068 | 0.781 | -0.288 | 0.232 | 0.026 | 0.915 | 0.052 | 0.834 | -0.009 | 0.972 |
| EN | 0.194 | 0.427 | -0.133 | 0.587 | 0.097 | 0.693 | 0.295 | 0.221 | -0.148 | 0.547 |
| AN | -0.438 | 0.060 | -0.274 | 0.256 | 0.118 | 0.632 | 0.052 | 0.832 | 0.161 | 0.510 |
| DAN | -0.324 | 0.175 | -0.324 | 0.176 | 0.162 | 0.506 | 0.021 | 0.933 | 0.280 | 0.246 |

NC: normal control; AEN: alloegocentric navigation; EN: egocentric navigation; AN: allocentric navigation; DAN: delayed allocentric navigation; BF: basal forebrain; EC: entorhinal cortex. *: *p* < 0.05. *P* values were adjusted for age, gender, years of education, total intracranial volume, and hippocampal volume.
