# Supplementary material for: Basal Forebrain Atrophy Is Associated With Allocentric Navigation Deficits in Subjective Cognitive Decline
Source: Front Aging Neurosci. 2021 Feb 15;13:596025. doi: 10.3389/fnagi.2021.596025 (PMC7917187; doi:10.3389/fnagi.2021.596025)
Supplement: Supplementary Box 1 — Subjective cognitive decline questionnaire. [file Data_Sheet_1.docx]

Supplementary Box 1

Items included in subjective cognitive decline questionnaire:

1. Do you think you have problems with your memory?

- Yes
- No

2. Do you have difficulty remembering a conversation from a few days ago?

- Yes
- No

3. Do you have complaints about your memory in the last 2 years?

- Yes
- No

4. How often is the following a problem for you: Personal dates?

- Always
- Sometimes
- Never

5. How often is the following a problem for you: Phone numbers you

use frequently?

- Always
- Sometimes
- Never

6. On a whole, do you think that you have problems remembering

things that you want to do or say?

- Yes
- No

7. How often is the following a problem for you: Going to the store and

forgetting what you wanted to buy?

- Always
- Sometimes
- Never

8. Do you think that your memory is worse than 5 years ago?

- Yes
- No

9. Do you feel you are forgetting where things were placed?

- Yes
- No

Total points:

Note:

Yes/Always: 1 point

Sometimes: 0.5 point

No/Never: 0 point
